# The EDA/EDAR/NF-κB pathway in non-syndromic tooth agenesis: A genetic perspective

**DOI:** 10.3389/fgene.2023.1168538

**Published:** 2023-04-03

**Authors:** Yanzi Gao, Xiaohui Jiang, Zhi Wei, Hu Long, Wenli Lai

**Affiliations:** ^1^ State Key Laboratory of Oral Diseases & National Clinical Research Center for Oral Diseases, West China Hospital of Stomatology, Sichuan University, Chengdu, China; ^2^ Department of Orthodontics, West China Hospital of Stomatology, Sichuan University, Chengdu, China; ^3^ Human Sperm Bank, Key Laboratory of Birth Defects and Related Diseases of Women and Children (Sichuan University), Ministry of Education, West China Second University Hospital, Sichuan University, Chengdu, China

**Keywords:** non-syndromic tooth agenesis (NSTA), hypohidrotic ectodermal dysplasia (HED), genetic basis, signaling pathway, EDA, EDAR, EDARADD

## Abstract

Non-syndromic tooth agenesis (NSTA) is one of the most common dental developmental malformations affected by genetic factors predominantly. Among all 36 candidate genes reported in NSTA individuals, *EDA*, *EDAR*, and *EDARADD* play essential roles in ectodermal organ development. As members of the EDA/EDAR/NF-κB signaling pathway, mutations in these genes have been implicated in the pathogenesis of NSTA, as well as hypohidrotic ectodermal dysplasia (HED), a rare genetic disorder that affects multiple ectodermal structures, including teeth. This review provides an overview of the current knowledge on the genetic basis of NSTA, with a focus on the pathogenic effects of the EDA/EDAR/NF-κB signaling pathway and the role of *EDA*, *EDAR*, and *EDARADD* mutations in developmental tooth defects. We also discuss the phenotypic overlap and genetic differences between NSTA and HED. Ultimately, this review highlights the importance of genetic analysis in diagnosing and managing NSTA and related ectodermal disorders, and the need for ongoing research to improve our understanding of these conditions.

## 1 Introduction

Tooth agenesis (TA), one of the most common dental developmental malformations in humans, negatively impacts aesthetics, mastication, and enunciation (Wong et al., 2014). TA can be divided into syndromic tooth agenesis (STA), which involves concomitant symptoms other than the congenital absence of teeth, and non-syndromic tooth agenesis (NSTA), which affects dentition solely. It has been known that NSTA is a multi-etiological developmental anomaly. Compared with exogenous factors, compelling evidence has indicated that genetic factors play a predominant role in the etiology of NSTA (Nieminen, 2009; Yin and Bian, 2015; Ye and Attaie, 2016). NSTA can be inherited in an autosomal or gonosomal pattern and occur sporadically or in families (Sarkar et al., 2014; Wang et al., 2016; Al-Ani et al., 2021; Albu et al., 2021; Asano et al., 2021; Khan et al., 2022). It has been confirmed that more than 80 genes were associated with TA (Yin and Bian, 2015), and 36 candidate genes have been identified in individuals with NSTA (Letra, 2022). Of these reported genes, *EDA*, *EDAR*, and *EDARADD* are candidate genes of both NSTA and STA (Ruiz-Heiland et al., 2016; Zhang et al., 2021a). Ectodysplasin-A (EDA), ectodysplasin-A receptor (EDAR), and EDAR-associated death domain (EDARADD), encoded by *EDA*, *EDAR*, and *EDARADD* respectively, play an important role in odontogenesis, regulating tooth number, crown shape, and enamel formation (Arte et al., 2013; He et al., 2013; Fons Romero et al., 2017). As members of the EDA/EDAR/NF-κB signaling pathway, alterations occurring in any part of the signaling can affect the transduction, contributing to NSTA potentially. The fact that mutations in EDAR result in an identical phenotype to the loss of function of EDA further supports that this pathogenic signaling is one of the causes of NSTA (Monreal et al., 1998).

**FIGURE 1:**
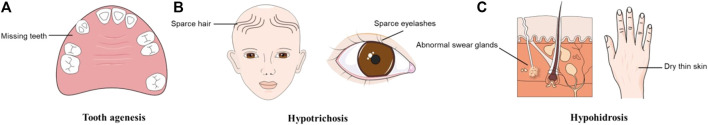
Typical symptoms of hypohidrotic ectodermal dysplasia (HED). **(A)**Tooth agenesis. **(B)** Hypotrichosis. **(C)** Hypohidrosis.

In addition to TA, changes in the EDA/EDAR/NF-κB signaling pathway are also involved in other ectodermal structure development disorders, leading to ectodermal dysplasia (ED) (Chassaing et al., 2006). Hypohidrotic ectodermal dysplasia (HED) is the most highly represented ED and is proven to be affected by mutations in *EDA*, *EDAR*, and *EDARADD* in most cases (Cluzeau et al., 2011; Martínez-Romero et al., 2019; Ahmed et al., 2021). HED is often characterized by TA, sparse hair and eyelashes (hypotrichosis), abnormal swear glands (hypohidrosis), and dry thin skin (Tariq et al., 2007; Mues et al., 2010; Bergendal et al., 2011) (Figure 1). Sharing a common genetic etiology, it was hypothesized that the distinction between STA and NSTA is not apparent (Mues et al., 2010; Zhang et al., 2011). Epigenetic regulation may explain this phenomenon, although further research is required to confirm this theory (Zhang et al., 2021a). The phenotypic overlap between NSTA and HED has led to confusion and controversy over the classification and diagnosis of these conditions. It underscores the need for a better understanding of their genetic and clinical features.

In this review, we will provide an overview of the current state of knowledge regarding the genetic basis of NSTA related to the EDA/EDAR/NF-κB pathway and its phenotypic overlap with HED, aiming at illustrating the importance of genetic analysis in the diagnosis of NSTA and related ectodermal disorders, and the need for ongoing research to improve our understanding of these conditions.

## 2 Genetic basis of the EDA/EDAR/NF-κB signaling pathway

### 2.1 The signaling mechanism

The *EDA*-encoded ligand EDA belongs to the tumor necrosis factor (TNF) ligand superfamily. During tooth development, EDA is continuously expressed in the dental epithelium from the epithelial thickening to the bud stage and then secreted into the mesenchyme, where it continues to be expressed until the end of the cap stage (Tucker et al., 2000). It has been established that EDA controls the formation of enamel knots, which operate as dental signaling centers, and facilitates communication between different epithelial compartments (Laurikkala et al., 2001; Häärä et al., 2012). EDA has 8 isoforms due to alternative splicing (Fan et al., 2008). However, only EDA-A1 (a 391-amino-acid protein) and EDA-A2 (a 389-amino-acid protein) have a receptor-binding domain among all isoforms (Kurban et al., 2010; Park et al., 2019; Liu et al., 2022). The EDA trimers in both isoforms are formed by three jelly-roll β-sandwich monomers (Hymowitz et al., 2003). EDA-A1 is a type II transmembrane protein with 4 functional domains: an N-terminal intracellular domain, a furin protease recognition sequence, a collagen-like repeat domain, and a C-terminal TNF homology domain (Ezer et al., 1999; Schneider et al., 2001; Mikkola and Thesleff, 2003; Andreoni et al., 2020). The C-terminal portion comprising the collagen and TNF homology domains cleaves off at the furin consensus site, releasing homotrimers that can bind to its particular receptor EDAR (Yan et al., 2000; Bodmer et al., 2002; Mikkola and Thesleff, 2003). EDA-A2, however, binding with its receptor X-linked ectodysplasin A (XEDAR), which is also encoded by EDAR, is not involved in TA (Newton et al., 2004; Mues et al., 2009). Although XEDAR can also recruit TRAF3 and TRAF6 to activate the NF-κB pathway (Verhelst et al., 2015), an intact EDA-A2/XEDAR signaling pathway cannot replace the blocked EDA/EDAR/NF-κB signaling pathway.

EDAR, a member of the TNF receptor superfamily, is a type I transmembrane protein containing 448 amino acids (Wang et al., 2020). EDAR is expressed in the dental epithelium at early stages and then in the enamel knot during the cap stage (Yamashiro et al., 2007). It has three cysteine-rich domains in the extracellular region (LBD), a transmembrane region, a death domain in its intracellular region, and a signal peptide (Kowalczyk-Quintas and Schneider, 2014; Zhang et al., 2021a; Yang et al., 2022). The homotrimers formed from EDA-A1 bind specifically to the LBD on the surface of dental epithelial cells and interact with each other through their death domains (Sadier et al., 2014). This interaction activates the recruitment of the EDARADD adaptor protein (Kumar et al., 2001). Subsequently, this process can recruit a member of the TNF receptor-associated factor (Traf) family into the signaling complex, which can recruit other molecules into the signaling complex (Courtney et al., 2005). The Traf family of scaffold proteins has six known members (Wajant et al., 2001), among them, Traf1, Traf2, Traf3, Traf4, and Traf6 are all expressed in the developing tooth germ, exhibiting dynamic spatiotemporal patterns (Courtney et al., 2005).

**FIGURE 2:**
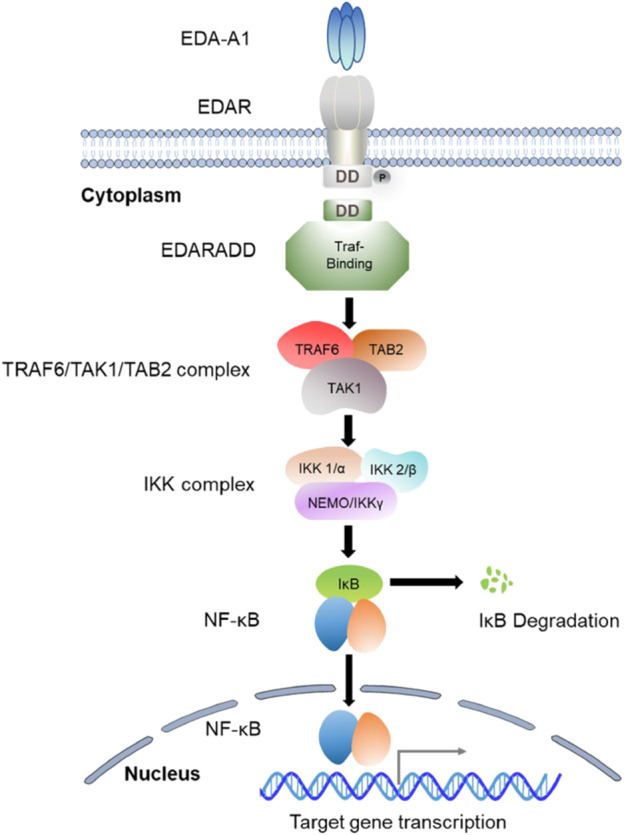
The signaling mechanism of the EDA/EDAR/NF-κB Pathway.

EDARADD is a 208-amino-acid protein that consists of a C-terminal death domain and an N-terminal Traf-binding consensus sequence (Kuramoto et al., 2011). The death domain participates in its self-association and the interaction with EDAR, and the Traf-binding consensus sequence combines with the TRAF6/TAK1/TAB2 complex to mediate the activation of a multimeric complex (IκB kinase (IKK)) and ultimately activates NF-κB (Cui and Schlessinger, 2006). As a scaffold molecule, the Traf is able to recruit the IKK complex, which begins the process of NF-kB activation (Courtney et al., 2005). The IKK complex is composed of two kinase subunits (IKK1/α and IKK2/β) and a structural component (NEMO/IKKγ) (Israël, 2000). Research has demonstrated that both the IKKα and β subunits exhibit catalytic activity, while NEMO/IKKγ is involved in the recruitment of IκBs (Barczewski et al., 2019). Moreover, IKKβ has been identified as the predominant kinase responsible for IκB phosphorylation (Yamada et al., 2020; Jimi and Katagiri, 2022). The phosphorylation of IκBs triggers their polyubiquitination, proteasomal degradation by the 26s proteasome, and eventually the release of NF-κB transcription factor into the nucleus (Chen and Greene, 2004). NF-κB is a homo or heterodimer that can be formed from different combinations of RelA (p65), c-Rel, RelB, p50/p105 (NF-κB1), and p52/p100 (NF-κB2) (Courtney et al., 2005; Yamada et al., 2020; Jimi and Katagiri, 2022). After the nuclear translocation of NF-κB, it induces the expression of genes essential for initiating and differentiating skin appendages such as hair, teeth, and sweat glands (Figure 2). Studies have suggested that NF-κB plays an essential role in regulating the expression of proteins related to amelogenesis and can impair the enamel formation (Liang et al., 2019; Yamada et al., 2020).

### 2.2 *EDA* mutations underlying NSTA

The EDA gene is located as a 425-kb segment on the long arm of the X-chromosome (Xq12-13.1) (Zonana et al., 1988). It can be inherited as XLR (OMIM 305100) or XLD (OMIM 313500). It was first discovered as a candidate gene in an NSTA individual in 2001 (Schneider et al., 2001) and was first identified in an X-linked inherited NSTA family in 2006 (Tao et al., 2006). To date, 45 mutations in *EDA* have been linked to NSTA (Schneider et al., 2001; Tao et al., 2006; Tarpey et al., 2007; Fan et al., 2008; Han et al., 2008; Li et al., 2008; Rasool et al., 2008; Mues et al., 2009; Song et al., 2009; Ayub et al., 2010; Mues et al., 2010; Arte et al., 2013; He et al., 2013; Nikopensius et al., 2013; Yang et al., 2013; Lee et al., 2014; Sarkar et al., 2014; Zhang et al., 2015; Gaczkowska et al., 2016; He et al., 2016; Shen et al., 2016; Bock et al., 2017; Martins et al., 2017; Yamaguchi et al., 2017; Zeng et al., 2017; Martínez-Romero et al., 2019; Parveen et al., 2019; Zhang et al., 2021a; Biedziak et al., 2022; Yu et al., 2022), with missense mutations accounting for the majority (39/45). In addition, deletion mutations (3/45), altered splicing (2/45), and non-sense mutation (1/45) were also found in NSTA cases.

Interestingly, we noted that nearly 80% of the mutations related to NSTA happened in the TNF homology domain of EDA, indicating that this region is crucial for EDA structure and functions. Specifically, the majority of the residues associated with NSTA were found close to the surface of the TNF homology domain (Yang et al., 2013). Previous studies have demonstrated that the position of amino acids in the TNF homology domain can affect the multimerization of EDA, leading to unnatural signaling (Song et al., 2009; Yang et al., 2013). The TNF homology domain has two distinct regions; one contains seven trimer-forming residues (H252, F302, Y304, Y347, C352, F379, and I383), and the other has five receptor binding residues (S275, I277, S319, 326E, and 328P). Specific amino acid changes in this region can cause the hydrogen bonds, hydrophilic or hydrophobic interaction, or electrostatic interactions between the monomers to be eliminated or damaged and the residues to be altered or reduced (Song et al., 2009; Zhang et al., 2015). According to 3D structural analysis, the stability of EDA homotrimers was partially disrupted by variations in the TNF domain, resulting in the inability of EDA to bind with EDAR (Elomaa et al., 2001; Tarpey et al., 2007; Li et al., 2008; Ayub et al., 2010; Lee et al., 2014). Another hotspot for EDA mutations is the collagen domain, which is also important for the function of EDA. It participates in the multimerization of EDA trimers, which is necessary for efficient signaling *via* high-valency receptor clustering (Mikkola and Thesleff, 2003). Thus, it was suggested that alterations in this region could inhibit multimerization of the TNF homology domain (Nikopensius et al., 2013). Two deletion mutations (c.612-629del; c.663-680del) were discovered in this region (Arte et al., 2013; Gaczkowska et al., 2016), and the deletions resulting in the shortening of the collagen domain ultimately affected its function. Moreover, mutations in the junction of transmembrane and extracellular domains changed the polarity of amino acids of EDA and affected its interactions with EDAR (Fan et al., 2008; Mues et al., 2009). The last mutant spot of EDA is the furin consensus site. The release of the TNF homology domain may be affected, and mutations in this region may impair proteolytic processing.

In conclusion, mutations in *EDA* can affect EDA/EDAR/NF-κB signaling pathway in the following ways: (Wong et al., 2014): aberrant multimerization altering the overall structure of EDA; (Ye and Attaie, 2016); Impairment of the EDA-EDAR binding activity; (Nieminen, 2009); abnormal transmembrane trafficking and proteolytic cleavage (Tao et al., 2006).

### 2.3 *EDAR* mutations implicated in the development of NSTA

The EDAR gene is a 425-kb segment located on chromosome 2q11-13. In total, 24 *EDAR* variants have been identified in NSTA since 2013 (Arte et al., 2013; Chen et al., 2017; Yamaguchi et al., 2017; Zeng et al., 2017; Jonsson et al., 2018; Mumtaz et al., 2020; Zhang et al., 2020; Zhang et al., 2021a; Zhang et al., 2021b; Biedziak et al., 2022; Yu et al., 2022; Zhao et al., 2022). As an integral component of the EDA/EDAR/NF-κB signaling pathway, EDAR binds to EDA through the extracellular LBD upstream and interacts with EDARADD through the intracellular death domain downstream. Consistent with this, the majority of the observed variation sites occurred in these two regions precisely, affecting either the upstream or the downstream. It was proposed that the mutant LBD can present conformational changes as a result of the substitution of amino acids, thus reducing its affinity with EDA subsequently (Zhang et al., 2020; Zhang et al., 2021a). Similarly, the mutant death domain could also cause the unstable structure of EDAR, reducing its ability to bind with EDARADD. As a result, the TRAF6/TAK1/TAB2 complex recruitment was decreased, which further affected the hydrolysis of NF-κB inhibitors and eventually reduced the activation of NF-κB (Sharp et al., 2015).

Similar to mutations in *EDA*, missense mutations accounted for the vast majority (21/24) of mutant types, while non-sense mutations made up a comparatively small proportion (3/24). However, no other types of mutations in *EDAR* have yet been described as NSTA-associated. By structural modeling analysis and molecular dynamics simulations, the structural dysfunction of missense mutant EDAR could be attributed to the following possible reasons: (Wong et al., 2014): alterations of the charge distribution of protein surface and declination of the strength and numbers of hydrogen bonds and hydrogen bonds; (Ye and Attaie, 2016); limited protein backbone motions trigger certain conformation changes of EDAR, making the protein unstable consequently; (Nieminen, 2009); loss of the attenuation of affinity with EDARADD (Zhao et al., 2022).

Regarding the non-sense mutations, it has been suggested that they can result in an early termination codon that causes the deletion of the wild-type *EDAR*’s residues. The first investigation into an NSTA-related non-sense *EDAR* mutation showed that the mutation (c.73C>T) truncated 424 amino acids of EDAR and eliminated the entire transmembrane domain and death domain (Zeng et al., 2017). The altered protein is, therefore, not anticipated to have any function. Likewise, the other two non-sense mutations (c.1302G>A; c.1072C>T), resulting in the deletion of certain residues, would prevent EDAR from binding to EDARADD (Mumtaz et al., 2020; Zhang et al., 2020).

### 2.4 *EDARADD* mutations as a contributing factor to NSTA

The EDARADD gene is a 423-kb segment located on chromosome 1q42.3-43. Despite the fact that only four NSTA-linked missense mutations in *EDARADD* have been reported (Bergendal et al., 2011; Arte et al., 2013; Gabrikova et al., 2016; Salvi et al., 2016; Martínez-Romero et al., 2019), the significance of EDARADD in tooth development is non-negligible. Mutant EDARADD can affect the signaling and lead to NSTA by losing its affinity for EDAR or reducing its capacity to bind to TRAF6. Compared with NSTA, however, more mutations of *EDARADD* were found in HED patients and usually with a more severe phenotype of the congenital tooth absence than non-syndromic ones. Nevertheless, it remains unknown why *EDARADD* mutations in NSTA individuals did not generate any ectodermal symptoms other than TA (Bergendal et al., 2011). It was proposed that the *EDARADD* inheritance pattern had an impact on the degree of EDARADD function loss (Asano et al., 2021). On the one hand, dominantly inherited *EDARADD* significantly diminished the ability to activate the downstream NF-κB due to the inability to bind with TRAF6. On the other hand, the recessively inherited mutant *EDARADD* only mildly diminished it on account of the mild reduction in the binding affinity with TRAF6 (Asano et al., 2021). However, a dominant inheritance pattern has also been found in the NSTA family with mutant EDARADD (Arte et al., 2013), thus the presence of the NSTA phenotype instead of the STA phenotype could not be explained by inheritance modes simply, and further investigations are needed.

## 3 Differences between HED and EDA/EDAR/NF-κB signaling pathway-related NSTA: A genetic insight

### 3.1 The phenotypic overlaps between HED and EDA/EDAR/NF-κB signaling pathway-related NSTA

HED is an uncommon congenital disorder characterized by aberrant ectodermal-derived structure development, which leads to abnormalities in skin appendages (Chassaing et al., 2006). As mentioned above, the EDA/EDAR/NF-κB signaling pathway is essential for ectodermal structure development. A report has revealed that four genes, including *EDA1*, *EDAR*, and *EDARADD*, are responsible for 90% of ED cases (Cluzeau et al., 2011). Additionally, mutations in *EDAR* account for 25% of non-EDA-related HED cases (Chassaing et al., 2006). There is a phenotypic overlap between HED and EDA/EDAR/NF-κB signaling pathway-related NSTA, as both conditions can present with missing teeth. Due to ongoing discoveries and advancements in genetic research, more and more scientists propose that HED and EDA-related NSTA are the same diseases with different degrees of severity (Mues et al., 2010; Zhang et al., 2011; Zeng et al., 2015; Gaczkowska et al., 2016). An attenuated phenotype has been regarded as a non-syndromic trait when the patient is affected by only one defective ectoderm-derived structure (Martínez-Romero et al., 2019; Wright et al., 2019). In other words, NSTA is presumably a variable expression of HED (Zhang et al., 2011). However, when concomitant ectodermal symptoms are too moderate to be recognized in clinical practice, patients can be misdiagnosed as NSTA (Chassaing et al., 2006; Tarpey et al., 2007; Bergendal et al., 2011; Plaisancié et al., 2013). Differentiating between HED and NSTA has implications for treatment strategies and long-term management. HED patients may require multidisciplinary care to address the various systemic manifestations, whereas NSTA patients may primarily require dental interventions. This has brought us to wonder where the boundary between HED and EDA/EDAR/NF-κB signaling pathway-related NSTA lies due to the same potential pathogenic signaling pathway. There is a growing body of evidence suggesting that the genetic underpinnings of HED and NSTA are distinct (Cluzeau et al., 2011; Arte et al., 2013). Understanding the genetic differences between HED and NSTA is crucial for genetic counseling and family planning.

### 3.2 Genetic differences between HED and EDA/EDAR/NF-κB signaling pathway-related NSTA

One possible explanation for mutations in EDA, EDAR or EDARADD genes resulting in the NSTA rather than the full-spectrum HED phenotype is that these genes may be expressed at higher levels during tooth development compared to other ectodermal appendages (Fournier et al., 2018). From the expression patterns, the observed distinct phenotype of hypodontia rather than the full HED phenotype is caused when mutations in *EDA* only partially disrupt the interaction of the EDA homotrimers and their target receptors, significantly affecting their function only in the dental tissues (Tarpey et al., 2007; Li et al., 2008; Song et al., 2009; Mues et al., 2010). Moreover, studies have shown that STA-related *EDA* mutations completely eliminate the capacity of mutant EDA proteins to bind to their receptors, whereas NSTA-related *EDA* mutations only decrease this ability (Mues et al., 2010). From the mouse rescue experiments, a small amount of recombinant EDA is sufficient for rescuing hair and salivary gland defects. In contrast, a relatively large amount of recombinant EDA is needed to save the number of teeth (Casal et al., 2007). This indicates that EDA signaling is required to develop ectodermal organs in a tissue-specific and dose-dependent manner (Mustonen et al., 2004; Casal et al., 2007). In all, mutations that entirely disrupt the EDA-EDAR interaction lead to severe HED phenotypes, whereas those causing only a partial weakening of the interaction give rise to mild non-syndromic manifestations (Yu et al., 2023).

It has been found that different mutation sites can have different impacts on the structure of proteins. From a systematic 3D conformation analysis, most of the residues associated with EDA-related NSTA are found near the surface of the TNF homology domain, while a large portion of residues associated with HED is buried in the interior of the domain (Yang et al., 2013). Although non-surface-exposed mutations were also found in NSTA (Rasool et al., 2008), it seems reasonable to postulate that mutations at sites buried in the interior could drastically alter EDA’s structure and might have more significant impact than mutations at regions close to the surface (Yang et al., 2013).

However, these hypotheses are still being investigated and may be subject to further refinement or revision as more research is conducted. The two conditions may represent a spectrum of severity rather than distinct diseases. Correct recognition of their overlapping symptoms and distinguishment from the genetic level is conducive to correct diagnosis and treatment.

## 4 Summary

The EDA/EDAR/NF-kB signaling pathway is involved in the development of ectodermal structures, including teeth, hair, and sweat glands. Therefore, defects in the EDA/EDAR/NF-kB pathway can disrupt this process and prevent the proper development of the teeth and other ectodermal organs. This review concludes the signaling mechanism of the pathway and provides a detailed summary for the first time of the molecular mechanisms of EDA, EDAR, and EDARADD, elucidating how gene mutations at different domains lead to protein alterations and ultimately result in the development of NSTA. Moreover, this review highlights the phenotypic overlap between EDA/EDAR/NF-κB signaling pathway-related NSTA and HED and provides novel insight to differentiate these conditions at the genetic level. By focusing on the genetic analysis of these disorders, the review emphasizes the importance of accurate diagnosis and management of NSTA and related ectodermal disorders. However, more research is needed to fully understand the mechanisms by which these mutations lead to the development of NSTA. By better understanding how this pathway works, researchers hope to develop new treatments and targeted therapies to address these conditions.
